# Improved Generative Adversarial Network for Super-Resolution Reconstruction of Coal Photomicrographs

**DOI:** 10.3390/s23167296

**Published:** 2023-08-21

**Authors:** Liang Zou, Shifan Xu, Weiming Zhu, Xiu Huang, Zihui Lei, Kun He

**Affiliations:** 1 School of Information and Control Engineering, China University of Mining and Technology, Xuzhou 221116, China; liangzou@cumt.edu.cn (L.Z.); sfxublues@cumt.edu.cn (S.X.); weimingzhu@cumt.edu.cn (W.Z.);; 2 Research Institute of Petroleum Exploration and Development, Beijing 100083, China; huangxiu1983@petrochina.com.cn

**Keywords:** coal photomicrographs restoration, super-resolution, generative adversarial net, wide residual block, 68T10, 97R40

## Abstract

Analyzing the photomicrographs of coal and conducting maceral analysis are essential steps in understanding the coal’s characteristics, quality, and potential uses. However, due to limitations of equipment and technology, the obtained coal photomicrographs may have low resolution, failing to show clear details. In this study, we introduce a novel Generative Adversarial Network (GAN) to restore high-definition coal photomicrographs. Compared to traditional image restoration methods, the lightweight GAN-based network generates more explicit and realistic results. In particular, we employ the Wide Residual Block to eliminate the influence of artifacts and improve non-linear fitting ability. Moreover, we adopt a multi-scale attention block embedded in the generator network to capture long-range feature correlations across multiple scales. Experimental results on 468 photomicrographs demonstrate that the proposed method achieves a peak signal-to-noise ratio of 31.12 dB and a structural similarity index of 0.906, significantly higher than state-of-the-art super-resolution reconstruction approaches.

## 1. Introduction

Coal is the mixture of organic macerals, including vitrinite, liptinite as well as inertinite, and inorganic minerals [1,2]. Analyzing coal photomicrographs and conducting maceral analysis provide valuable insights into the coal’s composition, quality, and potential applications. Advanced microscopy techniques, such as scanning transmission electron microscope [3], enable the acquisition of high-quality photomicrographs. Nevertheless, these precise instruments require skilled operators and often have limited availability in most laboratories due to the high cost. Conventional microscopes or older photomicrographs often suffer from limited resolution, particularly in laboratories with constrained funding. Re-acquiring photomicrographs with advanced microscopes involves substantial expenses and labor-intensive procedures, such as sieving, molding, and polishing. Recently, with the rapid development of computer vision, machine learning methods were introduced to identify maceral groups in given photomicrographs [4,5,6]. To effectively explore these low-resolution photomicrographs, this study proposes a novel strategy to enhance the resolution of low-resolution photomicrographs. The approach involves utilizing an improved generative adversarial network (GAN) to obtain high-quality images, allowing for precise maceral identification and analysis. This method presents a promising solution to overcome the limitations of conventional techniques and improve the performance of coal photomicrographs analysis.

The purpose of super-resolution (SR) is to convert low-resolution image with coarse details into corresponding high-resolution image with better visual quality and refined details. Single-Image Super-Resolution (SISR) is an important branch of SR, which aims to determine the mapping function between a low-resolution image and a high-resolution image, and reconstruct the corresponding high-resolution image. Although promising results have been achieved, it remains a challenging problem in computer vision. Filtering approaches, such as linear, bicubic interpolation, and Lanczos re-sampling, are classical methods of enhancing resolution, which can reconstruct images quickly and straightforwardly. Freedman et al. introduced a filter based on local self-similarity observations to search for similar patches, whereas its performance is suboptimal in clustered regions with fine details [7]. These filtering methods tend to oversimplify the Single-Image Super-Resolution (SISR) problem and result in a loss of image details [8]. Additionally, they may lead to over-smooth texture in the reconstructed images.

With the rapid advance of machine learning, deep learning has attracted more and more attention in computer vision and medical signal analysis, and is widely used in the super-resolution field [9,10]. Super-resolution CNN (SRCNN) employs neural network to solve SISR problem, which demonstrates strong capability of learning rich features from big data in an end-to-end manner [8]. However, its shallow architecture restricts its performance. Very Deep Super-Resolution (VDSR), with 20 residual layers, enhances the performance of super-resolution image reconstruction, whereas it consumes much more computational cost [11,12]. Fast Super-Resolution Convolutional Neural Network (FSRCNN) has a relatively shallow network structure consisting of four convolution layers and one deconvolution layer [13]. It was demonstrated to have faster speed and better reconstructed image quality than the SRCNN. Although significant improvement in terms of accuracy and reconstruction speed were achieved, one critical problem for that period was that the resulted high-resolution images always have poor visual quality, especially for the cases with large upscaling factors. In addition, the loss function of these methods has largely focused on minimizing the mean squared error (MSE) between the restored image and the ground truth. These methods aim to enhance the Peak Signal-to-Noise Ratio (PSNR), and may ignore high-frequency information, leading to over-smoothed results.

To address this concern, the researchers mainly improve the reconstruction performance from the prospective of loss function and network structure. Ledig et al. developed a novel framework for Super-Resolution based on the Generative Adversarial Network (SRGAN), and proposed perceptual loss function instead of traditional MSE loss [14]. It calculates the difference between the generated and real images in feature space based on pretrained VGG, thus preserving more realistic details and textures. The experimental results indicate that SRGAN achieved finer texture details even with large up-scaling factors in comparison with SRCNN. The core concept of Multi-Agent Diverse GAN (MAD-GAN) involves simultaneous training of multiple generators, wherein each generator is tasked with producing a set of related yet not entirely consistent samples, thus facilitating more efficient data processing [15]. During the training process, the discriminator assesses the images generated by the generators and assigns a reward score to each generator, which serves as an indicator of the quality of the generated samples. Consequently, the optimization objective for the generators encompasses not only the minimization of differences with real samples but also the maximization of diversity among the generated samples. This char acteristic endows MAD-GAN with the ability to not only generate high-quality samples but also maintain sample diversity, effectively preventing the generation of excessively similar samples. Wang et al. made significant improvements to the key components of SRGAN by introducing residuals in the dense blocks. This facilitates the flow of fine-detail features to deep layers of the network. Additionally, they further preserved the details’ information by removing batch normalization [16]. Compared with SRGAN, the proposed enhanced SRGAN (ESRGAN) achieved better perceptual quality with more realistic and natural textures for the visual sense. RFB-ESRGAN emerged as the winning solution for the image super-resolution reconstruction task in the NTIRE 2020 challenge [17]. It effectively integrates the distinctive traits of the RFB-Net and ESRGAN models, while introducing receptive field blocks into the feature extraction network to enhance the capture of global and local features within the images. This incorporation of receptive field blocks notably benefits the handling of objects or textures at various scales.

While deep learning models have shown remarkable performance in Single-Image Super-Resolution (SR), the networks proposed recently for general images are not suitable for the reconstruction of coal photomicrographs. The deep neural networks proposed in this research perform well with common images, such as those in the DIV2K and Flickr2K datasets [18]. DIV2K is the foremost dataset extensively employed for training super-resolution reconstruction models, renowned for its high quality. It encompasses 800 training images, 100 validation images, and 100 test images. On the other hand, Flickr2k constitutes a vast extended dataset with 2650 2K images originating from the renowned image-sharing platform Flickr, a subsidiary of Yahoo. However, their application to coal photomicrographs can lead to the undesired presence of artifacts, causing performance issues. Coal photomicrographs predominantly consist of black and gray colors. Although they do exhibit differences across various macerals, the level of complexity in terms of details and textures is not as high as that found in natural images. Considering the unique characteristics of coal photomicrographs, we specifically designed a novel framework based on improved GAN to enhance the resolution of these images without unwanted artifacts.

The developed super-resolution model is trained with faster speed and fewer parameters in comparison with the state of the art. The main contributions of the proposed method are as follows:

1. Given the unique characteristics of coal photomicrographs, which set them apart from traditional images, we have specifically designed a lightweight generative adversarial network to enhance the resolution of these photomicrographs. Experimental results indicate that the proposed method surpasses state-of-the-art GAN-based methods.
2. We propose a novel residual block called the Wide Residual Block (WRB), designed to enhance the neural network’s non-linear fitting ability and feature extraction capabilities while minimizing computational load. By integrating WRBs into the network architecture, the modified network is able to produce smoother and more continuous restoration effects without introducing artifacts, outperforming networks utilizing traditional residual blocks.
3. We utilize a pyramid attention block that can be seamlessly integrated into existing super-resolution networks. This block significantly improves the performance of super-resolution models by enhancing their capability to capture important feature relationships across multiple scales. The related codes and dataset are publicly available at the following website: https://github.com/Jackson-LIMU/SR-IGAN (accessed on 30 January 2023).

The rest of this paper is organized as follows. Section 2 introduces the architecture of the networks. Section 3 presents the details and evaluation metrics of the experiments. Section 4 shows the experiment results of the proposed methods and comparison with existing methods. Section 5 presents the conclusion of this paper.

## 2. Network Architecture

### 2.1. The Overall Structure of the Proposed Method

**Figure 1:**
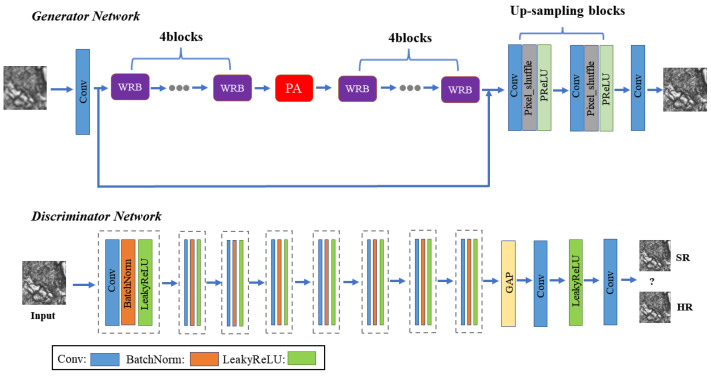
The detailed architecture of the proposed generator and discriminator network.

The standard GAN includes a generative module (*G*) and a discriminative module (*D*), which are trained simultaneously with contradictory objectives [19,20]. The *G* tries to generate new samples with the inputs, and the *D* aims to classify its inputs as real or generated. These two modules are trained until the Nash equilibrium. In this work, the generator tries to create a super-resolution image and fool the discriminator. The discriminator is trained to distinguish whether the input high-resolution image is a real image or an image generated by the generator. The overall architecture of the proposed method is illustrated in Figure 1.

The input low-resolution image is initially processed by a convolutional layer with 64 filters (kernel size: 9 × 9), followed by ReLu [21] as the activation function. Then, we employ eight wide residual blocks (WRBs) to serve as the backbone of the generator, with a pyramid attention (PA) block embedded after the fourth WRB. There are two up-sampling blocks, where each one contains a convolution layer which enlarges the number of input channels by four times, and a Pixel-Shuffle layer [22] as well as Parametric ReLU (PReLU). The discriminator consists of eight convolutional blocks, and the number of channels increases from 64 to 512, as in the VGG network. The convolutional layer is configured with a stride of 2, leading to a downsizing of the output feature map. Each convolution is followed by batch normalization and LeakyReLU (α=0.2). A Global Average Pooling (GAP) layer follows the final convolutional block, averaging the pixel values in a feature channel. This output directly links to the final classification block. The GAP acts as a regularizer, aiding in the mitigation of overfitting while also reducing the overall count of parameters in the model. The final classification block contains two convolutional layers and one LeakyReLU layer, where the channels are first changed from 512 to 1024 and end up with one. The resulted feature map is processed by the sigmoid activation function to classify the input image. Besides, the detailed architecture of the proposed network in terms of the kernel size and padding specifications is shown in Table 1 (generator) and Table 2 (discriminator).

### 2.2. WRB Block

**Figure 2:**
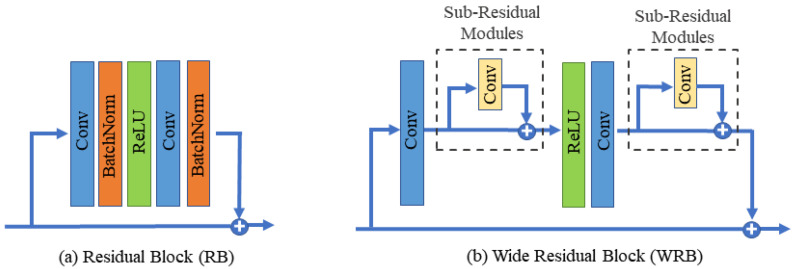
The comparison between (**a**) traditional residual block with batch normalization and (**b**) wide residual block, which replaces BN with a subresidual module.

Deep network architectures typically possess superior feature learning capabilities and often outperform shallow networks [23]. However, their training can be more challenging, and they require a substantial volume of samples. Without adequate training data, these networks may fail to generalize to the test set, despite performing well on the training set. The residual network was introduced to tackle this issue. Its use of skip connections enables the feasible design of very deep networks [24]. Furthermore, batch normalization (BN) is typically applied immediately after the convolutional layer and prior to the activation in the standard residual block. BN addresses the internal covariate shift problem by normalizing layer inputs. Concurrently, it enhances the network’s generalization capability and expedites the training process to some extent [25], as shown in Figure 2a. In the context of image super-resolution, the output images are required to have same color, contrast, and brightness as the input ones. However, batch normalization may inadvertently alter the image’s contrast. When an image passes through a BN block, its color distribution is normalized, potentially disrupting the original contrast information. Moreover, BN layers can introduce artifacts in the case of a deep network trained under a GAN framework [16].

In this study, we propose a novel wide residual block without a BN layer to address these concerns, as illustrated in Figure 2b. We replace the BN layers in the standard residual block with two subresidual modules to widen the feature extraction network. Each subresidual structure comprises only one convolutional layer, with no inclusion of an activation function or batch normalization. This approach reduces the computational load of the convolutional layer, thus further enhancing the network’s training speed. The mathematical expression for the subresidual module is presented as follows:

(1) $$\begin{array}{c} R\left(a\right)=C_{1}\left(a\right)+a \end{array}$$

where *a* represents the input to the subresidual module, R(a) denotes its output, and $C_{1}$ represents the subresidual convolution, whose kernel size is set to 7 × 7 and the padding is set to 3 in this study.

The wide residual module employs a combination of long and short connections. Specifically, the input of the wide residual block is directly linked to that block’s output, enabling subsequent blocks to directly access the input information of the preceding layer. Long connections typically serve to fuse high-level semantic information with low-level detail information. Meanwhile, the short connection in the wide residual block directly links the input and output of the subresidual structure, further expanding the model’s receptive field. In addition, this structure aids in extracting deeper image features while preserving the input information, thereby enhancing the representation of deep semantic information. Suppose *b* represents the input to the WRB module and WRB(b) denotes the output of this module. The mathematical expression of the WRB module is as follows:

(2) $$\begin{array}{c} WRB\left(b\right)=R(C_{2}\left(\sigma \left(R\left(C_{2}\left(b\right)\right)\right)\right))+b \end{array}$$

where *R* represents the subresidual module mentioned earlier, σ represents the Rectified Linear Unit (ReLU) activation function, and $C_{2}$ denotes a convolution layer with a kernel size of 3 × 3 and padding of 1.

### 2.3. Pyramid Attention Module

The self-similarity that small but similar patterns tend to occur at different locations and scales is widely explored via non-local operations in image restoration field. Although deep-learning-based SR methods have made great progress, many of them fail to fully exploit the self-similarities, since the self-attention modules process information at the same scale. The pyramid attention network (PAN) was first proposed to increase the receptive field and improve the accuracy in classifying small objects in semantic segmentation [26]. It was demonstrated to be capable to capture the global contextual information and exploit long-range dependence from a multi-scale feature pyramid [26,27]. In this work, we introduce a pyramid attention module (PAM) to evaluate correlation among features across multiple specified scales by searching over the entire pyramid target and regions. The new response from fusing non-local multi-scale information intuitively contains richer and more credible information than single-scale information.

**Figure 3:**
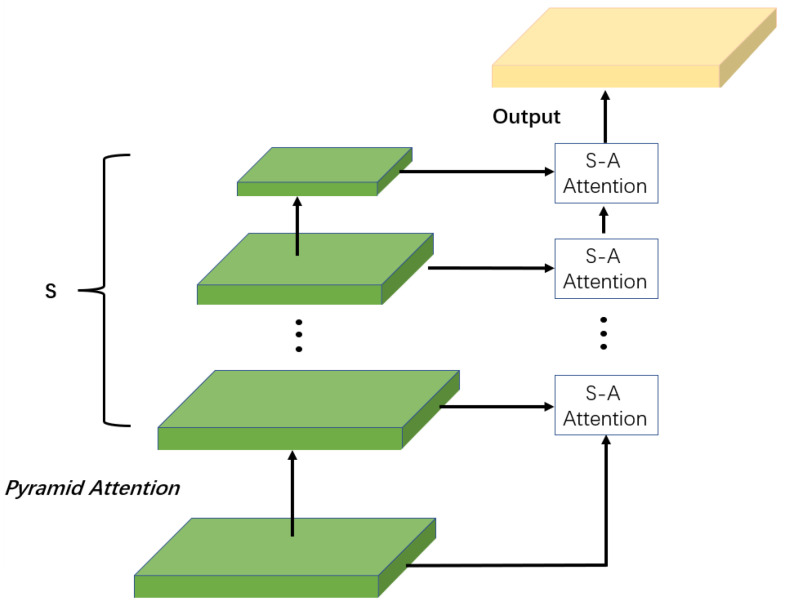
Pyramid attention captures multi-scale feature correspondences by employing a series of Scale Agnostic attention.

The pyramid attention captures correlations across multiple scales in a bottom-up manner, as shown in Figure 3, where Scale Agnostic (SA) attention is applied to two adjacent scale features successively. Unlike classic non-local attention, which computes pixel-wise feature correlation, SA attention is a self-attention operation of block-wise matching which is able to generate better restoration images. The main reason is that block-matching introduces an extra constraint on nearby pixels, and thus, is able to differentiate highly relevant correspondences while suppressing unrelated ones. Specifically, we employ bicubic interpolation to build feature pyramids with five different scales. The four bicubic interpolations for scaling are at sizes of 0.9 times, 0.8 times, 0.7 times, and 0.6 times the dimensions of the original input image. The multi-scale attention architecture is able to capture non-local feature correspondences across multiple specified scales without scale restriction. In addition, it fuses adjacent scale information, which contributes to extracting context features more precisely. In addition, region-to-region matching imposes additional similarity constraints on the neighborhood, and thus, the module is able to effectively pick out highly correlated dependencies.

**Figure 4:**
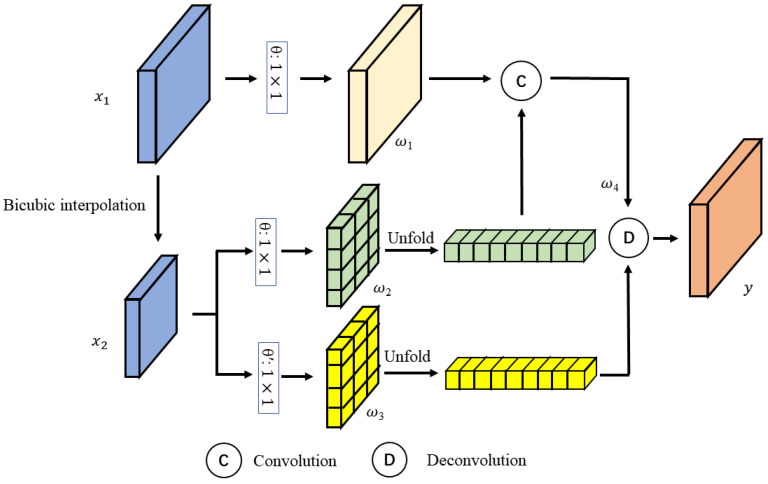
The SA attention structure.

The schematic diagram of the SA attention structure is depicted in Figure 4, where $x_{1}$ represents the feature map output from the previous SA module and $x_{2}$ is obtained by down-sampling $x_{1}$ through bicubic interpolation. Initially, $x_{1}$ is input to a convolutional layer denoted as θ, where the output channel is half of the input channel, resulting in $\omega_{1}$. Subsequently, $x_{2}$ was input to two separate convolutional layers denoted as θ and $\theta^{\prime }$, where $\theta^{\prime }$ has the same number of input and output channels. The resulting feature maps $\omega_{2}$ and $\omega_{3}$ are utilized for feature reconstruction and feature transformation, respectively. Moreover, $\omega_{2}$ acts as the kernel weight for performing convolution with $\omega_{1}$ after the unfold operation. The kernel size for the unfold operation is 3 × 3, and the dilation rate is 1 × 1. The output $\omega_{4}$ is input into the deconvolutional layer with kernel weight of the unfolded $\omega_{3}$, resulting in the final output *y*.

### 2.4. Loss Function

The loss function is employed to measure the difference between the reconstructed images and high-resolution (HR) ground truth, and optimize the model iteratively. The Mean Squared Error (MSE) loss function is a commonly-used loss function to evaluate the pixel-wise matching in previous super-resolution studies, which is calculated as follows:

(3) $$\begin{array}{c} L_{MSE}=\frac{1}{WH}\sum_{w=1}^{W}\sum_{h=1}^{H}{\left(I_{HR}^{w,h}-G{\left(I_{LR}\right)}^{w,h}\right)}^{2} \end{array}$$

where $I_{HR}$ and $I_{LR}$ represent high-resolution and low-resolution images, respectively, *W* and *H* are the width and height of the high-resolution image, and *G* represents the generator. The variables *w* and *h* represent the pixel coordinates of corresponding positions in the image.

Although being widely used in the previous studies, the MSE loss based on pixel difference between reconstructed image and ground truth cannot wholly represent the quality of super-resolution reconstruction [28]. The methods based on MSE loss are able to provide high PSNR, whereas the outputs always have over-smooth textures, leading to perceptually unsatisfying solutions. In order to address this concern, Ledig et al. introduced the VGG loss to measure the difference between the feature representation of the reconstructed image and ground truth, avoiding the over-smooth results [14]. In this paper, we utilize a pre-trained VGG19 neural network [29] to formulate the perceptual loss. Pre-training VGG19 involves training the model on a large dataset (e.g., ImageNet) to learn informative features from natural images before incorporating it as a component of the loss function in a specific task, such as super-resolution. This loss function quantifies perceptual similarity by extracting feature maps from both the real and reconstructed images, and computing their Euclidean distance in feature space, which is calculated as follows:

(4) $$\begin{array}{c} L_{VGG}=\frac{1}{WH}\sum_{w=1}^{W}\sum_{h=1}^{H}(\Phi \left(I_{HR}^{w,h}\right)-\Phi {\left(G{\left(I_{LR}\right)}^{w,h}\right)}^{2} \end{array}$$

where Φ represents the feature extraction function corresponding to the first 16 layers of the VGG19 architecture.

In the process of image restoration, a little bit of noise on the image may exert great damage on the restoration result, since super-resolution restoration algorithms might amplify the noise. To address this issue, we employ the total variation (TV) loss as the regular item to maintain the smoothness of the image. The difference in the values of adjacent pixels in the image can be resolved to a certain extent by reducing the TV loss. The TV loss is defined as the following formula:

(5) $$\begin{array}{c} L_{TV}=\sum_{i,j}{\left({\left(x_{i,j-1}-x_{i,j}\right)}^{2}+{\left(x_{i+1,j}-x_{i,j}\right)}^{2}\right)}^{\frac{\beta }{2}} \end{array}$$

where the difference between each pixel and the adjacent pixel is calculated on the right, as well as the pixel beneath it. The β is set to be 2, by default.

In this study, a weighted sum of Mean Squared Error (MSE) loss, perceptual loss, and Total Variation (TV) loss is used to create the content loss, which comprehensively evaluates the quality of the reconstructed image from the perspectives of pixel, feature maps, and noise, which is denoted as follows:

(6) $$\begin{array}{c} L_{Content}^{SR}=L_{MSE}+\lambda_{1}L_{VGG}+\lambda_{2}L_{TV} \end{array}$$

where $\lambda_{1}$ and $\lambda_{2}$ represent weight parameters. Inspired by SRGAN, we empirically set the perceptual loss weight $\lambda_{1}$ and the TV loss weight $\lambda_{2}$ to be 0.006 and 2 $\times \;10^{-8}$, respectively.

Furthermore, the adversarial loss [14] is employed to train the generator, thereby fostering the generation of increasingly realistic samples to confound the discriminator. The adversarial loss formula is represented by:

(7) $$\begin{array}{c} L_{Gen}^{SR}=\sum_{n=1}^{N}-logD(G\left(I_{LR_{n}}\right)) \end{array}$$

where *N* represents the total number of samples, *n* represents the *n*-th sample, and *D* represents the discriminator.

The final loss function $L^{SR}$ is composed of a weighted sum of the content loss $L_{Content}^{SR}$ and the adversarial loss $L_{Gen}^{SR}$:

(8) $$\begin{array}{c} L^{SR}=L_{Content}^{SR}+\lambda_{3}L_{Gen}^{SR} \end{array}$$

where $\lambda_{3}$ represent weight parameter, which is set to be 0.001, drawing upon pertinent experience.

## 3. Experimental Setup

### 3.1. Experiment Details

We acquired 468 high-resolution coal photomicrographs using the polarizing microscope, and 48 of them were randomly selected as the validation dataset. The remaining images were shuffled and divided as the training dataset and test dataset with a ratio of 8 to 2. The original size of photomicrographs is 2580 × 1944. Inputting whole images into the network would significantly increase the computational resource requirements and training time. Therefore, we perform random cropping on the coal photomicrographs to obtain a corresponding small-sized image. The use of random cropping strategy ensures that the feature information input to the network for the same original image may vary in each epoch, which helps the model generalize well to new data. The evaluation metrics are solely used to assess the super-resolution reconstruction performance of the cropped images, i.e., the results for individual patches. As shown in Table 3,we experimented with different patch size (e.g., 512 × 512, 256 × 256, 128 × 128, and 64 × 64), and found that while larger patch sizes might enhance overall quality, they do not necessarily improve finer details. Conversely, smaller patch sizes, such as 64 × 64, enhance finer details but limit the receptive field. Therefore, we made a trade-off between the finer details and the receptive field of the image, and set crop size to be 256 × 256 pixels.

**Figure 5:**
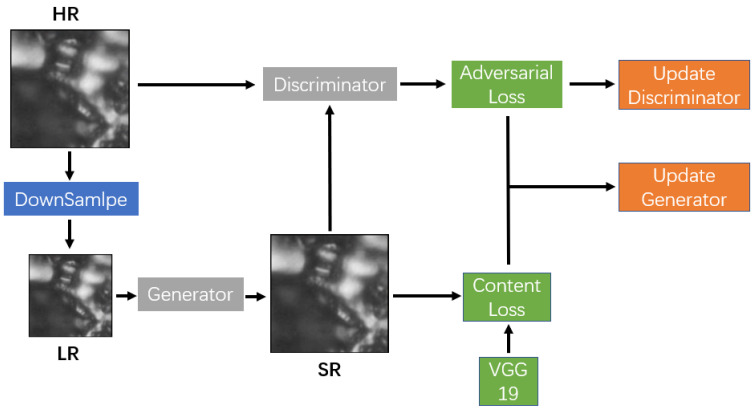
Training process for our modified GAN, which completely demonstrates how the network works during each iteration.

Then, we downsampled these patches four times via bicubic interpolation to obtain low-resolution (LR) images. These LR images were fed into the generator and the reconstructed SR images were fed to the discriminator. We employed the combination of content loss and adversarial loss to update generator. The discriminator was updated by adversarial loss. The training process is shown in Figure 5.

All the experiments were implemented on a workstation with NVIDIA RTX-3090-Ti GPU. We trained the model for about 350 epochs with a batch size of 64, and each epoch contains six iterations with a learning rate of $10^{-3}$. We employed Adam optimizer [30] with $\beta_{1}=$ 0.9 and $\beta_{2}=$ 0.99 to optimize the generator and the discriminator networks alternately.

### 3.2. Evaluation Indices

We employ two objective image quality assessment (IQA) indices [31], namely peak signal-to-noise ratio (PSNR) and structural similarity index metric (SSIM), to evaluate the quality of the reconstructed photomicrographs, which are widely used in the filed of SR. PSNR is a pixel-based metric of image quality and is defined via the maximum pixel value (MaxValue) and the mean squared error between the reconstructed images and the ground truth, as follows:

(9) $$\begin{array}{c} PSNR=10\log_{10}\frac{MaxValue^{2}}{MSE} \end{array}$$

where the MaxValue mostly equals to 255 provided the bit depth is 8 bits. It should be mentioned that this quality metric is not enough because the SR image might not be visually similar to that of the ground truth image, whereas PSNR is high. We introduce the SSIM [32] to measure the structural similarity between images in terms of the contrast, luminance, and structural details, defined as:

(10) $$\begin{array}{c} SSIM=\frac{\left(2\mu_{SR}\mu_{HR}+C_{1}\right)\left(\sigma_{SRHR}+C_{2}\right)}{\left(\mu_{SR}^{2}+\mu_{HR}^{2}+C_{1}\right)\left(\sigma_{SR}^{2}+\sigma_{HR}^{2}+C_{2}\right)} \end{array}$$

where $\mu_{SR}$ and $\mu_{HR}$ represent the average gray value of the reconstructed SR image and ground truth, respectively, $\sigma_{SRHR}$ is the covariance of the reconstructed SR image and ground truth, and $\sigma_{SR}^{2}$ and $\sigma_{HR}^{2}$ is the corresponding variance, respectively. In summary, PSNR and SSIM evaluate the quality of a image in two aspects, namely computer vision and human perception of structural information.

## 4. Experiment Results

### 4.1. Qualitative Results

**Figure 6:**
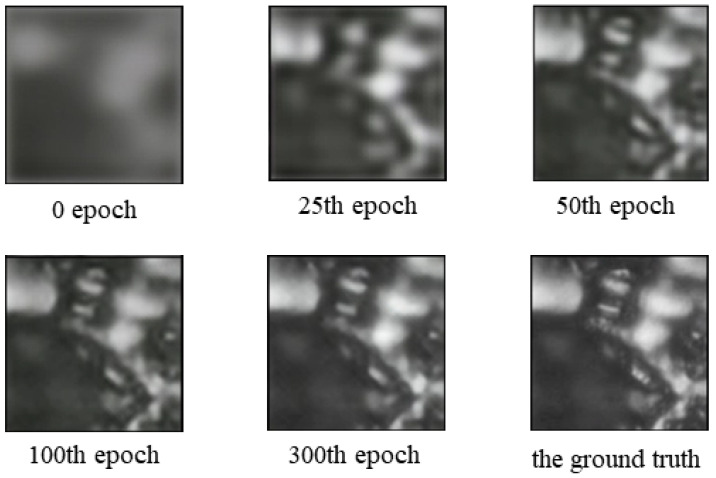
Coal photomicrographs super-resolution images produced by 4× up-scales using our proposed method at the 0, 25th, 50th, 100th, and 300th epoch. The last image is the ground truth.

**Figure 7:**
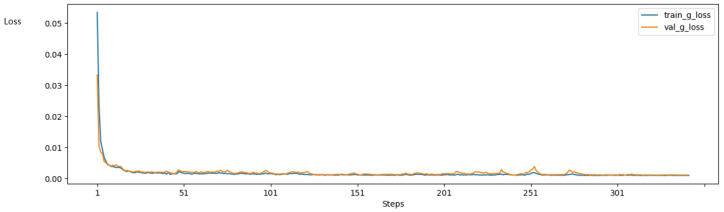
Line chart of loss during training and validating process, represented by blue and yellow curves, respectively.

To reveal the training results intuitively, we show an illustrative example of the reconstructed SR images generated by the proposed network after 0, 25, 50, 100, and 300 epochs. As we can see from Figure 6, the quality of an image gradually improves as time goes on. In the first few epochs, the network converges quickly, which leads to a visually obvious change in the output SR images. By the 100th epoch, the generated output becomes similar to the ground truth. The training and validating curves are shown as Figure 7. The training loss decreases with a rapid speed at the very first epochs and then declines slowly.

**Figure 8:**
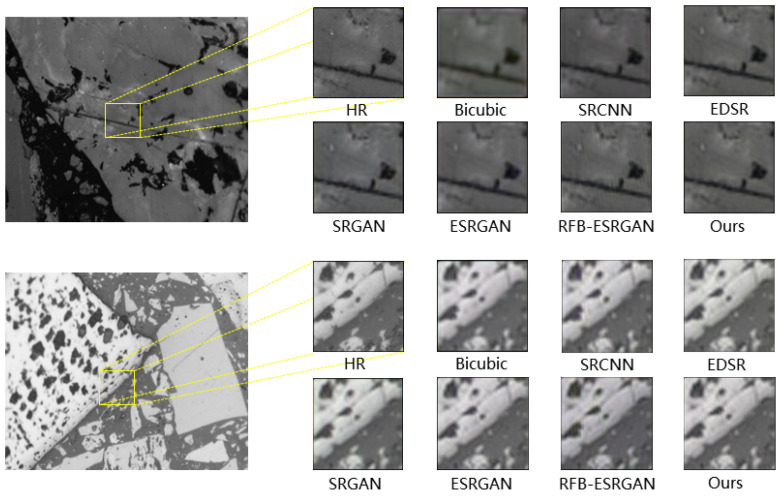
The reconstruction results of Bicubic interpolation, SRCNN, SRGAN, EDSR, ESRGAN, RFB-ESRGAN and our method, and the corresponding reference HR image.

We further compare the proposed method over coal photomicrographs with both traditional and state-of-the-art super-resolution reconstruction methods, including Bicubic interpolation [33], SRCNN [8], SRGAN [14], EDSR [34], ESRGAN [16], and RFB-ESRGAN [17]. Two illustrative examples are shown in Figure 8. It is demonstrated that the proposed method is able to generate more detailed textures in comparison to other methods. SRCNN and EDSR fail to produce enough details, causing the blur of the reconstructed SR images in comparison with GAN-based architectures. Although deeper networks tend to provide better performance in reconstructing common images (such as natural images), the models with complex structures and enormous parameters, such as SRGAN and RFB-ESRGAN, may generate overfitting results over the coal photomicrographs, since the textures and details of photomicrographs are not as fickle as common images. The previously proposed GAN-based models, such as SRGAN, may arouse unpleasant artifacts during the training.

### 4.2. Quantitative Results

We employ PSNR and SSIM as the evaluation indices to evaluate the performance of various SR methods, and the higher values of them demonstrate better reconstruction quality. We download the source codes of SRCNN, EDSR, SRGAN, ESRGAN, and RFB-ESRGAN from the authors’ homepage and re-train/evaluate these networks with the utilized dataset, including 336 photomicrographs. The results over the test set with 84 photomicrographs are shown in Table 4. As we can see from Table 4, the proposed method outperforms others with the highest PSNR and SSIM of 31.1210 dB and 0.9055, respectively. EDSR achieves the second-best PSNR of 30.4251 dB, while ESRGAN gets the second-best SSIM of 0.8986. Compared with SRGAN, the ESRGAN employs a residual-in-residual dense block without batch normalization and is able to generate more detailed structures, and thus, with a higher PSNR of 30.2009. Shang et al. applied receptive field block (RFB) to enhance the features’ discriminability and proposed the enhanced ESRGAN, namely RFB-ESRGAN. The corresponding parameters are significantly increased from 3,028,931 to 12,590,999, whereas the SSIM is decreased a bit from 0.8986 to 0.8910. The proposed method has similar number of parameters as EDSR and SRGAN, while obtaining significantly better performance, achieving a balance between the complexity of the network and performance. Leveraging the benefit of a relatively compact model parameter size, this approach is highly suitable for practical deployment, facilitating its seamless integration into web-based platforms for efficient image reconstruction. It is worth noting that the model presented in this study demonstrates an inference time of 0.125 seconds per image, which is lower than RFB-ESRGAN but higher than SRGAN and ESRGAN. Nevertheless, this processing time is in an acceptable range for the reconstruction of coal photomicrographs, where real-time reconstruction is not a critical requirement. The primary objective of this study is obtaining high-definition images with preserved details.

### 4.3. Ablation Study

**Figure 9:**
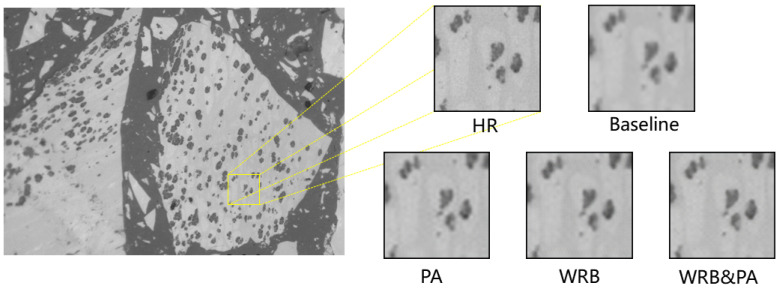
Comparison of ablation experiment results of a sample image from the test dataset.

In order to explore the effect of each module in the proposed architecture, we gradually modify the baseline model and evaluate its performance. For a fair comparison, the experimental configurations are kept the same across the baseline model and its variants. The overall comparison is illustrated in Table 5. We first replace the WRB with the conventional residual block, shown in Figure 2a, and remove the PA module in the proposed network, denoted as the baseline model. The baseline model achieves a PSNR of 29.9731 dB and SSIM of 0.8902, and the performance is relatively low. Subsequently, we replaced the traditional residual blocks in the baseline model with WRB and observed a significant improvement in PSNR and SSIM. This demonstrates the effectiveness of the proposed WRB. The WRB enhances the network’s non-linear fitting ability with 2.42% more parameters. We further evaluate the performance when the pyramid attention module is added on the baseline model. The PSNR and the SSIM are improved by 1.1479 dB and 0.0153, respectively, in comparison with the baseline model. It illustrates the effectiveness of PA module, which is capable of extracting high-level features to guide the weighting of low-level features without too much memory or computation burden. The proposed network with both WRB and PA module obtains the best performance in terms of both PSNR and SSIM, with 3.56% more parameters than the baseline model. Figure 9 demonstrates the super-resolution reconstruction results of the baseline model and its variants.

In addition, we consider three typical positions of PA module: after the first WRB block, after the fourth WRB block (in the middle of the network), and after the last WRB block. Table 6 demonstrates the performance in terms of PSNR and SSIM when the PA-net is placed at different positions within the generator network. We can see that the best performance is achieved by inserting the PA-net after the fourth WRB block.

## 5. Conclusions

Macerals, organic components present in coal, represent different types of plant materials transformed to varying degrees during coal formation. By analyzing coal photomicrographs and conducting maceral analysis, researchers and industry professionals gain valuable insights into coal’s composition, quality, and potential applications. As a result, coal photomicrograph analysis plays a critical role in assessing coal quality and advancing environmentally friendly mining practices. However, obtaining high-resolution coal photomicrographs is a cumbersome and costly process. To effectively explore the information from low-resolution photomicrographs, we propose a lightweight network designed to enhance the resolution of coal photomicrographs using an improved GAN-based super-resolution method. We propose a novel architecture called wide residual block with subresidual modules to replace the BN layers in conventional residual block. The removal of BN layers contributes to performance improvement, particularly in preventing the distortion of the image’s contrast. In comparison with the recently proposed GAN-based SR strategies, this architecture not only simplifies the generator network but also avoids unpleasant artifacts introduced by BN. In addition, we embed a five-level pyramid attention block in the middle of the generator to adaptively capture the long-range correlations between features. The module is fully differentiable and can serve as a common building block of SR networks to enhance image restoration performance. Moreover, we introduce a global average pooling layer prior to the last second convolutional layer of the discriminator to avoid the overfitting problem. We employ an additional loss function, referred to as TV-loss, to suppress noise during the training process. Collectively, these improvements contribute to the generation of images with more natural textures, finer sharpness, and intricate details. We evaluate the performance of the proposed method and the state of the art’s on 84 coal photomicrographs. The experimental results, both quantitative and qualitative, validate the effectiveness of our proposed approach.

It should be noted that the proposed method still has a few limitations. The main focus is on whether the high-resolution images generated by the generator comply with the geological rules. In addition, more photomicrographs are required to evaluate the generalization ability of the proposed method. All of these aspects require further validation through real-world applications. To address these concerns, in the future, we plan to invite experienced geologists to conduct subjective evaluation tests (MOS testing) on the generated coal photomicrographs to assess their perceptual quality. Additionally, we aim to incorporate domain knowledge into the super-resolution reconstruction network to provide superior performance and ensure that the reconstruction results are comprehensible to humans.

## Figures and Tables

**Table 1:** Generator framework details.

Layer	Kernel Size
Conv-first	9 × 9 × 64, padding 4
WRB-pre (×4)	3 × 3 × 64, 7 × 7 × 64, 3 × 3 × 64, 7 × 7 × 64
PA	/
WRB-post (×4)	3 × 3 × 64, 7 × 7 × 64, 3 × 3 × 64, 7 × 7 × 64
UpsampleBLock1	3 × 3 × 256
UpsampleBLock2	3 × 3 × 1024
Conv-last	9 × 9 × 3, padding 4

**Table 2:** Discriminator framework details.

Layer	Kernel Size and Stride
Convolution-1	3 × 3 × 64
Convolution-2	3 × 3 × 64, stride 2
Convolution-3	3 × 3 × 128
Convolution-4	3 × 3 × 128, stride 2
Convolution-5	3 × 3 × 256
Convolution-6	3 × 3 × 256, stride 2
Convolution-7	3 × 3 × 512
Convolution-8	3 × 3 × 512, stride 2
GAP	/
Convolution-9	1 × 1 × 1024
Convolution-10	1 × 1 × 1

**Table 3:** The performance metrics, PSNR and SSIM, were evaluated on the test set using images of different sizes as inputs to our model.

Input Sizes	PSNR (dB)	SSIM
64 × 64	30.4171	0.9074
128 × 128	31.0275	0.9023
256 × 256	31.1210	0.9055
512 × 512	32.0591	0.8810

**Table 4:** Performance comparison in terms of the PSNR, SSIM, parameters, and inference time.

Methods	PSNR (dB)	SSIM	Parameters	Inference Time for Each Image(s)
Bicubic [33]	29.1734	0.8254	None	0.071
SRCNN [8]	29.8132	0.8796	69,251	0.089
EDSR [34]	30.4251	0.8901	925,080	0.084
SRGAN [14]	29.9607	0.8897	734,219	0.104
ESRGAN [16]	30.2009	0.8986	3,028,931	0.090
RFB-ESRGAN [17]	30.4116	0.8910	12,590,999	0.148
The proposed method	31.1210	0.9055	760,328	0.125

**Table 5:** The PSNR and SSIM results of different modifications to the network. We use WRB block and PA block to confirm their effect.

Methods	PSNR	SSIM	Parameters
Baseline	29.9731	0.8902	734,219
Use WRB Block	30.6882	0.8997	752,005
Use PA Block	30.4737	0.8917	742,408
Use WRB & PA Block	31.1210	0.9055	760,328

**Table 6:** The PSNR and SSIM of different position of PA block to the module.

Position	PSNR	SSIM
after the first WRB	30.9437	0.9018
after the fourth WRB	31.1210	0.9055
after the last WRB	31.0836	0.9031

## Data Availability

The dataset is available upon request from the authors via email at liangzou@cumt.edu.cn.
